# miR-1908: a microRNA with diverse functions in cancers and non-malignant conditions

**DOI:** 10.1186/s12935-022-02709-5

**Published:** 2022-09-13

**Authors:** Soudeh Ghafouri-Fard, Tayyebeh Khoshbakht, Bashdar Mahmud Hussen, Mohammad Taheri, Majid Samsami

**Affiliations:** 1 Department of Medical Genetics, School of Medicine, Shahid Beheshti University of Medical Sciences, Tehran, Iran; 2 Men’s Health and Reproductive Health Research Center, Shahid Beheshti University of Medical Sciences, Tehran, Iran; 3 Department of Pharmacognosy, College of Pharmacy, Hawler Medical University, Erbil, Kurdistan Region Iraq; 4 Center of Research and Strategic Studies, Lebanese French University, Erbil, Kurdistan Region Iraq; 5 Urology and Nephrology Research Center, Shahid Beheshti University of Medical Sciences, Tehran, Iran; 6 Institute of Human Genetics, Jena University Hospital, Jena, Germany; 7 Cancer Research Center, Shahid Beheshti University of Medical Sciences, Tehran, Iran

**Keywords:** miR-1908, Cancer, Bipolar disorder

## Abstract

MicroRNAs (miRNAs) are small-sized transcripts with about 22 nucleotide length. They have been shown to influence almost every aspect of cellular functions through regulation of expression of target genes. miR-1908 is a miRNA with diverse roles in human disorders. This miRNA is encoded by *MIR1908* gene on chr11:61,815,161–61,815,240, minus strand. Expression assays have confirmed dysregulation of miR-1908 in cancer-derived cell lines in addition to biological samples obtained from patients affected with cancer. In most assessed cell lines, miR-1908 has an oncogenic role. However, this miRNA has been shown to act as a tumor suppressor in chordoma, lung cancer and ovarian cancer. In addition, several lines of evidence have shown involvement of this miRNA in the pathoetiology of bipolar disorder, myocardial infarction, obesity, renal fibrosis, rheumatoid arthritis and scar formation. In the current review, we elucidate the results of diverse studies which evaluated participation of miR-1908 in these conditions.

## Introduction

microRNAs (miRNAs) are a group of regulatory non-coding RNAs with sizes about 22 nucleotides [1]. In multicellular organisms, miRNAs affect both the stability and translation of mRNAs, thus participating in modulation of gene expression at post-transcriptional level. RNA polymerase II-mediated transcription of miRNAs leads to production of capped and polyadenylated primary transcripts, which are then, cleaved by a specific type of ribonuclease III enzymes. This enzymatic process results in production of stem-loop structures with approximate size of 70 nucleotides. These so-called precursor miRNAs are subjected to another round of cleavage by the cytoplasmic Dicer ribonuclease. The mature miRNA produced by these two rounds of processing is assimilated into a RNA-induced silencing complex (RISC). RISC identifies target mRNAs via a base pairing process resulting in suppression of mRNA translation or its destabilization [2]. miRNAs contribute in diverse biological processes for example cell proliferation, differentiation and apoptosis, thus contributing in the pathoetiology of diverse disorders [3, 4].

miR-1908 is encoded by *MIR1908* gene on chr11:61,815,161-61,815,240, minus strand. The stem loop sequence of this miRNA is as follows: CGGGAAUGCCGCGGCGGGGACGGCGAUUGGUCCGUAUGUGUGGUGCCACCGGCCGCCGGCUCCGCCCCGGCCCCCGCCCC (https://www.mirbase.org/cgi-bin/mirna_entry.pl?acc=MI0008329).

This miRNA contains some single nucleotide polymorphisms (SNPs). Genome wide association studies (GWAS) and evaluation of human regulatory elements (enhancers and promoters) have indicated association between these SNPs and red blood cell distribution width, serum metabolite levels, neuroimaging measurement, brain volume measurement, asthma, bipolar disorder, and response to carboplatin (https://www.genecards.org/cgi-bin/carddisp.pl?gene=MIR1908). Thus, this miRNA is regarded as a candidate gene for several human phenotypes and disorders. Expression assays have also confirmed abnormal expression levels of miR-1908 in cancer-derived cell lines as well as biological samples obtained from patients affected with cancer. In addition, several lines of evidence have shown involvement of this miRNA in the pathoetiology of bipolar disorder, myocardial infarction, obesity, renal fibrosis, rheumatoid arthritis and scar formation. In the current review, we elucidate the results of diverse studies which evaluated participation of miR-1908 in these conditions. The reason of selection of this miRNA was its newly identified roles in diverse cancers, particularly its opposite roles in different contexts.

### The impact of miR-1908 in the carcinogenesis based on cell line studies

Experiments in two breast cancer cells have indicated the role of miR-1908-3p in enhancement of cells proliferation, migration and invasion [5]. Further evaluations in these context have potentiated ID4, LTBP4, GPM6B, RGMA, EFCAB1, ALX4, OSR1 and PPARA as targets of this miRNA [5].

In cervical cancer cell lines, expression of miR-1908 has been found to be upregulated. Over-expression of miR-1908 has augmented growth and invasion of cervical carcinoma cells. Consistently, miR-1908 silencing has led to opposite effect. In silico and functional studies have validated interaction between miR-1908 and HDAC10. Besides, ectopic expression of HDAC10 in cervical cancer cells could reverse the effect of miR-1908 to some extent. Taken together, miR-1908 increases aggressive behavior of cervical cancer cells through targeting HDAC10 [6].

**Fig. 1:**
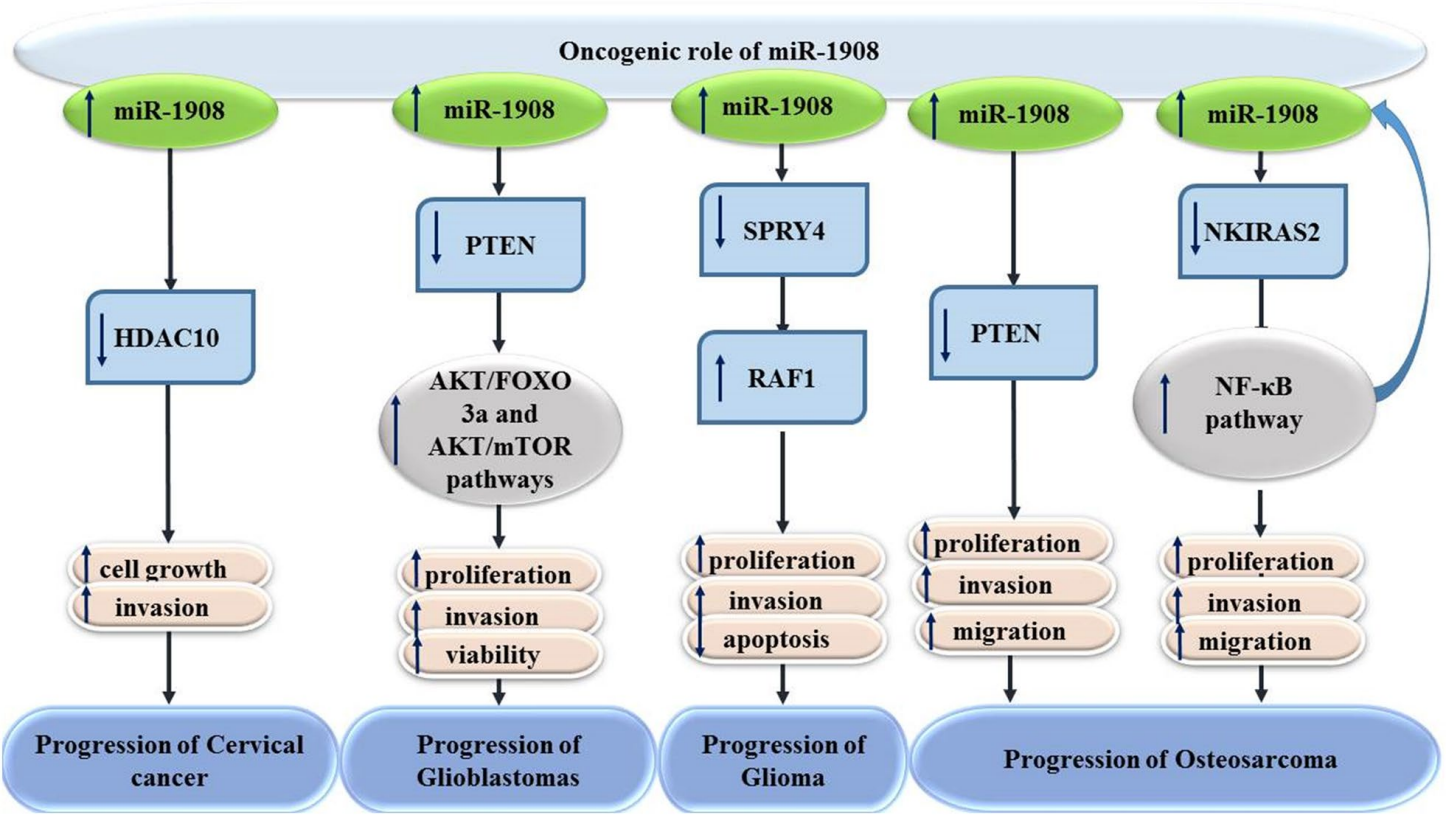
Oncogenic role of miR-1980

Up-regulation of miR-1908 in glioblastoma cells has enhanced anchorage-independent growth. This miRNA could suppress PTEN expression through binding with its 3’-UTR. Thus, miR-1908 has an oncogenic role in glioblastoma through inhibition of PTEN pathway [7]. Another study in glioma has shown that miR-1908 has a role in enhancement of proliferation and invasion, as well as suppression of apoptosis through regulation of SPRY4/RAF1 axis. In silico analyses has indicated involvement of miR-1908 in the regulation of pathways related with cell proliferation, invasion and apoptosis. Up-regulation of miR-1908 has induced anti-apoptotic effects in glioma cells via reducing expression levels of Bax. SPRY4 as one of validated miR-1908 targets has interactions with the pro-oncogene RAF1. Up-regulation of miR-1908 has led to down-regulation of SPRY4 expression and up-regulation of RAF1 [8]. Figure 1 shows the molecular axes mediating the oncogenic roles of miR-1908 in a number of cancers.

**Fig. 2:**
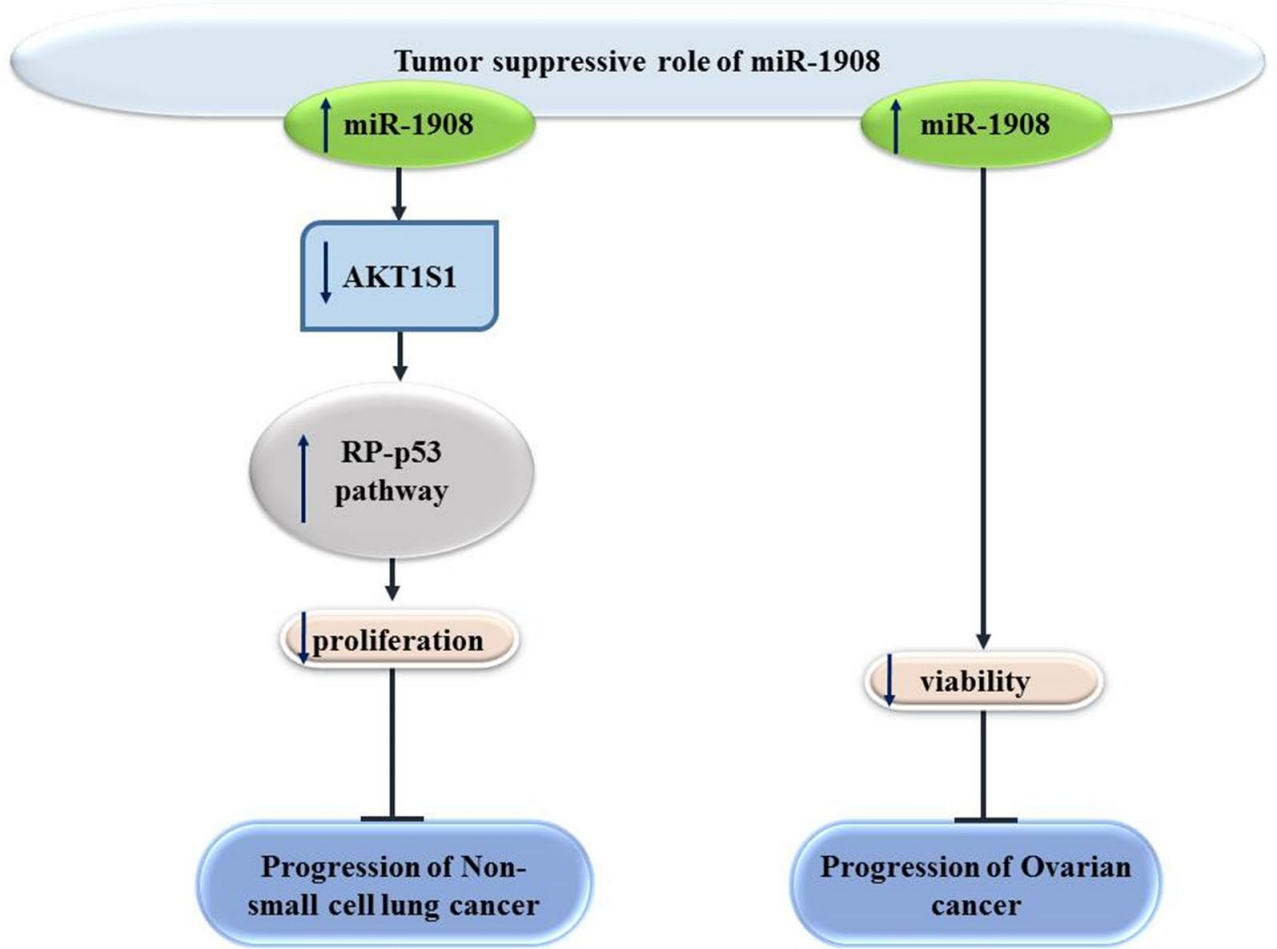
Tumor suppressor role of miR-1980

Contrary to the bulk of evidence in the above-mentioned types of cancers, miR-1908 has been shown to induce tumor suppressor impacts in ovarian cancer and lung cancer (Fig. 2). An in vitro study in non-small cell lung cancer has indicated down-regulation of miR-1908. miR-1908 mimics could reduce proliferation of these cells. Furthermore, RP-p53 pathway has been shown to be activated by miR-1908 mimics. The suppressor of the RP-p53, AKT1S1 has been found to be targeted by miR-1908 [9].

Table 1 summarizes the results of in vitro studies on the role of miR-1908 in the carcinogenesis.

**Table 1:** Function of miR-1908 in cancers based on studies in cell lines (∆: knock-down or deletion)

Tumor type	Interactions	Cell lines	Function	Exact role	References
Breast cancer	ID4, LTBP4, GPM6B, RGMA, EFCAB1, ALX4, OSR1 and PPARA	MCF-10A, MCF-7, MDA-MB-231	↑↑ miR-1908-3p: ↑ proliferation, migration and invasion	Oncogenic	[5]
Cervical cancer	HDAC10	Ca-Ski, SiHa, C-4 I and End1/E6E7	↑↑ miR-1908: ↑ cell growth and invasion	Oncogenic	[6]
Glioblastoma	PTEN, AKT/FOXO3a and AKT/mTOR pathways	A127, SW1783, U87, U373, LN-229, SW1088, Hs683, HFU251, SNB19, T98G, 1228 and 802	∆ miR-1908: ↓ Proliferation, viability, invasion and sphere formation	Oncogenic	[7]
Glioma	SPRY4/ RAF1 axis	U251	↑↑ miR-1908: ↑ proliferation, invasion and ↓ apoptosis	Oncogenic	[8]
Nasopharyngeal carcinoma	–	P109CD4 + TIL and P125CD8 + TIL cell lines	Hsa-miR-1908 was found to be over-expressed in TW03 (EBV +) or TW03 (EBV −) cells	–	[10]
Non-small cell lung cancer	AKT1S1 and RP-p53 pathway	HBE, SK-MES-1, A549 and NCI-H460	↑↑ miR-1908: ↓ Proliferation via targeting AKT1S1 ∆ miR-1908: ↑ Proliferation	Tumor suppressor	[9]
Osteosarcoma	SRSF3/miR-1908-5p/NKIRAS2 axis and NF-κB pathway	U2OS	↑↑ miR-1908–5: ↑ proliferation, migration, and invasion via targeting NKIRAS2 ↑↑ NF-κB pathway: ↑ expression of miR-1908-5p ∆ SRSF3: ↓ expression of miR-1908-5p via blocking transactivation of NF-κB	Oncogenic	[11]
	PTEN	143B, U-2 OS, MG-63 and Saos-2, and hFOB 1.19	↑↑ miR-1908–5: ↑ proliferation, migration, and invasion	Oncogenic	[12]
Ovarian cancer	–	A2780, and SK‐OV‐3	↑↑ miR-1908–5: ↓ cell viability	Tumor suppressor	[13]
Prostate cancer	SRM	22Rv1, PC3 and PC‐3 M‐luc‐C6	miR-1908/ SRM axis could control secretion of EVs in prostate cancer	–	[14]

### Function of miR-1908 in non-malignant disorders based on studies in cell lines

A single study in the context of bipolar disorder has stated that a number of validated targets of miR-1908-5p, namely DLGAP4, GRIN1, STX1A, CLSTN1 and GRM4 are involved in the regulation of glutamatergic synapses in neurons. Besides, in silico assessments have also confirmed inverse correlation between expression of miR-1908-5p and these synaptic targets in many regions of human brain. Expression levels of miR-1908-5p in normal human neural progenitor cells have been surged following chronic treatment with valproate. However, treatment of these cells with lithium has not affected expression of this miRNA. Most notably, valproate has reduced expression of this miRNA in neural progenitor cells originated from fibroblasts of a patient with bipolar disorder. Cumulatively, miR-1908-5p has been suggested to contribute in the pathogenesis of bipolar disorder [15].

Another study has indicated that over-expression of miR-1908 can improve cardiac function, decrease fibrosis of myocardial cells and decrease TGF-β1 and Smad2/3 levels. TGF-β1 has been shown to be targeted by miR-1908. In fact, miR-1908 inhibits expression of Smad2/3 via TGF-β1 [16].

Levels of miR-1908 have been shown to be elevated in the course of adipogenesis of human multipotent adipose-derived stem cells. Up-regulation of miR-1908 in these cells could inhibit adipogenic differentiation and enhanced proliferation of cells, demonstrating the effect of this miRNA in the differentiation and metabolism of adipocytes and pathoetiology of obesity [17]. Table 2 shows function of miR-1908 in non-malignant disorders based on studies in cell lines.

**Table 2:** Function of miR-1908 in non-malignant disorders based on studies in cell lines (∆: knock-down or deletion SD: Sprague–Dawley, RA: Rheumatoid arthritis, NPCs: neural progenitor cells)

Disease type	Interactions	Cell lines	Function	References
Bipolar disorder	DLGAP4, GRIN1, STX1A, CLSTN1 and GRM4	control and bipolar patient iPS cell lines	Valproate: ↑ miR-1908-5p expression in normal NPCs and ↓ miR-1908-5p expression in NPCs of patients with bipolar disorder	[15]
Myocardial infarction	TGF-β1 and Smad2/3	Cardiac fibroblasts from SD neonatal rats	miR-1908 was found to inhibit the Smad2/3 expression via targeting TGF-β1	[16]
Obesity	–	hMADS cells and HPA-v	↑↑ miR-1908: ↑ proliferation and ↓ adipogenic differentiation A high level of miR-1908 was observed during the adipogenesis	[17]
Obesity	IL-6, TNF-α, leptin and resistin	Human visceral preadipocytes	Levels of miR-1908 expression were found to be increased during the differentiation of human preadipocytes into adipocytes, and be regulated by adipokines	[18]
Renal fibrosis	TGF-β1, smad2/3 and MMP-2	Human primary renal interstitial cells	↑↑ miR-1908: ↓ expressions of TGF-β1, smad2/3 and MMP-2, and ↓ renal fibrosis process	[19]
Rheumatoid arthritis	HOTTIP/miR-1908–5p/ STAT3 axis	Rheumatoid arthritis synovial fibroblasts	↑↑ HOTTIP (which sponges miR-1908): ↑ inflammation in RA	[20]
Scar formation post-burn wound healing	Ski	Tissues from the dermis of six patients with hypertrophic scars	↑↑ miR-1908: ↑ fibrosis and scar formation ∆ miR-1908: ↓ fibrosis and inflammation	[21]

### Animal studies on the role of miR-1908 in cancers and non-malignant conditions

Few animal studies have assessed function of miR-1908 in animal models. Two studies have confirmed the oncogenic roles of miR-1908 in glioblastoma [7] and osteosarcoma [12] (Table 3).

**Table 3:** Function of miR-1908 in cancer based on studies in animal models

Tumor Type	Animal models	Results	References
Glioblastoma	4–6 week-old male BALB/c nude mice	↑↑ miR-1908: ↑ tumor volume, tumor weight and tumor growth	[7]
Osteosarcoma	4–6 week-old male BALB/c athymic nude mice	↑↑ miR-1908: ↑ tumor volume and tumor weight	[12]

Moreover, contribution of miR-1908 in the pathogenesis of myocardial infarction, renal fibrosis and scar formation has been verified in animal models (Table 4).

**Table 4:** Function of miR-1908 in non-malignant conditions based on studies in animal models (SD: Sprague–Dawley)

Disease Type	Animal models	Results	References
Myocardial infarction	8–10-week-old male SD rats	miR-1908 expression was reduced at 4 weeks after myocardial infarction ↑↑ miR-1908: ↓ myocardial fibrosis, and TGF-β1 and Smad2/3 levels	[16]
Renal fibrosis	renal fibrosis mouse models	↑↑ miR-1908: ↓ renal fibrosis	[19]
Scar formation post-burn wound healing	male SD rats	↑↑ miR-1908: ↑ scar formation	[21]

### Tumor suppressor versus oncogenic roles of miR-1908 based on studies in clinical samples

Expression of miR-1908-3p has been reported to be elevated in breast cancer tissues and sera of these patients compared with corresponding controls. Besides, its expression has been higher in tissue samples of young patients and HER2-positive samples compared with samples obtained from older patients and HER2-negative tumors, respectively. Similarly, serum level of miR-1908-3p has been higher in younger patients compared with elder ones. Most notably, higher levels of miR-1908-3p target genes have been correlated with better clinical outcomes in this type of cancer [5].

In cervical cancer samples, the expression of miR-1908 has been inversely correlated with transcript levels of HDAC10. Notably, over-expression of HDAC10 has been associated with better prognosis of cervical cancer [6].

In glioblastoma, miR-1908 expression has been significantly higher in the patients with high risk of tumor recurrence compared to those with lower risk of recurrence. Moreover, over-expression of miR-1908 has been correlated with poor survival of patients. Taken together, miR-1908 has been regarded as a putative biomarker for estimation of risk of recurrence in patients with glioblastoma [7]. Another study has shown down-regulation of SPRY4 as one of validated targets of miR-1908 in glioma samples. Markedly, down-regulation of SPRY4 has been correlated with short survival time in these patients [8].

In ovarian cancer, miR‐1908‐5p has been among miRNAs that predict progression free survival of patients [13].

In brief, dysregulation of miR-1908 has been associated with poor prognosis in cervical cancer, glioblastoma, osteosarcoma and ovarian cancer (Table 5).

**Table 5:** Results of studies that reported dysregulation of miR-1908 in clinical samples from cancers (ANCTs: adjacent non-cancerous tissues, OS: overall survival, DFS: disease-free survival, TNM: tumor node metastasis, PFS: progression-free survival, HGSOC: high‐grade serous ovarian carcinoma)

Tumors	Samples	Expression (Tumor vs. Normal)	Kaplan–Meier analysis (impact of miR-1908 up-regulation)	Univariate/ Multivariate cox regression	Association of miR-1908 expression with clinical characteristics	Reference
Breast cancer	TCGA dataset 50 pairs of tumor tissues and ANCTs 60 breast cancer patients compared to 60 healthy controls	Up (Younger breast cancer patients and those with HER2-positive tumors had a higher levels of this miRNA)	–	–	age and her-2 status	[5]
Cervical cancer	GSE63514 36 pairs of tumor tissues and ANCTs	up	Shorter OS	–	–	[6]
Chordomas	3 chordomas tissues and 3 notochord tissues	Down	–	–	–	[22]
Glioblastoma	47 glioma patients and five normal brain samples	Up	Shorter OS and DFS	–	–	[7]
Glioma	GEO and TCGA databases	Up	Shorter OS and DFS	–	–	[8]
Nasopharyngeal carcinoma	10 NPC patients and 10 healthy controls	Up	–	–	–	[10]
Osteosarcoma	212 pairs of tumor tissues and ANCTs	Up	Shorter OS	–	Metastasis, poorer chemotherapy response and greater extent of recurrence	[23]
Osteosarcoma	46 osteosarcoma samples and 9 normal muscle samples	Up	Shorter OS	–	Advanced TNM stage and tumor growth	[12]
Ovarian cancer	TCGA dataset (491 patients with OC)	Down	Better OS and DFS	miR-1908 expression level was found to be an independent predictor of OS of patients with OC and its expression was associated with age	–	[24]
Ovarian cancer	175 patients with HGSOC GSE106817	Down in HGSOC	–	miR-1908-5p was found to be an indicator of PFS	–	[13]
Ovarian cancer	15 platinum-sensitive ovarian cancer patients and 15 platinum-resistant ovarian cancer patients	Up in platinum-resistant patients	–	–	–	[25]

### Dysregulation of miR-1908 in clinical samples in non-malignant conditions

miR-1908 has been among miRNAs being under-expressed in depression episodes of the bipolar disorder compared with remission state. This study has suggested miR-1908 one of the most promising miRNAs for diagnosis of depression phase of this disorder [26]. Besides, miR-1908 has been among differentially expressed miRNAs between pre-stroke and post-stroke phases in diverse subtypes of ischemic stroke. Moreover, miR-1908 showed significant diagnostic values in both large artery atherosclerosis and lacunar infarct patients [27]. Table 6 shows the results of research projects that revealed dysregulation of miR-1908 in clinical samples from non-malignant conditions.

**Table 6:** Dysregulation of miR-1908 in clinical samples from non-malignant conditions (UP: unipolar disorder, BP: bipolar disorder)

Disease type	Samples	Expression (Disease vs. Normal)	References
Bipolar disorders	17 UP and 15 BP patients	Down in bipolar disorder patients in comparison to remission phase	[26]
Ischemic stroke	13 normal control subjects (NCs), 23 cardioembolism (CARD), 26 cases of large artery atherosclerosis (LAA), 27 cases of lacunar infarct (LAC), and 11 cases of stroke of undetermined etiology (SUE) 20 NCs, 28 CARD, 23 LAA, 18 LAC, and 16 SUE	Down in LAA, LAC, and SUE patients but not in CARD patients	[27]
Obesity	16 human adipose tissues of obese cases and 12 matched normal tissues	Down in subcutaneous, up in visceral tissues of obese patients	[17]
Scar formation post-burn wound healing	Wound tissue and adjacent normal dermal Tissues from 46 patients with deep second-degree burns and hypertrophic scarring	Up in burn-wounded skin compared with adjacent normal skin, down when the wound was healing and then increased during scar development	[21]

Wohlers et al. have analyzed miRNA expression quantitative trait loci among 115 GWAS regions linked with inflammatory disorders. Their comprehensive functional fine-mapping has demonstrated two independent GWAS regions associated with autoimmune diseases risk SNPs with important impacts on miRNA expression. These regions have been shown to influence expression of miR-1908-5p and have been related to SNPs associated with Crohn's disease (rs102275) and rheumatoid arthritis (rs968567) [28].

## Discussion

miR-1908 has been shown to contribute in the pathoetiology of several disorders through targeting important signaling pathways, namely PTEN, AKT/FOXO3a and AKT/mTOR, SPRY4/ RAF1, RP-p53 and NF-κB pathways. The majority of studies have indicated an oncogenic role for miR-1908. However, this miRNA has been shown to act as a tumor suppressor in chordoma, lung cancer and ovarian cancer.

Notably, dysregulation of miR-1908 has been associated with poor prognosis in cervical cancer, glioblastoma, osteosarcoma and ovarian cancer. However, the diagnostic role of miR-1908 has not been fully investigated in the context of cancer.

miR-1908 has been among miRNAs that could be used as diagnostic markers in two neuropsychiatric conditions, i.e. bipolar disorder and ischemic stroke. This miRNA has a putative function in fibrotic conditions as well. This function is most probably exerted through modulation of TGF-β signaling.

Taken together, miR-1908 participates in the pathogenesis of different types of cancers, as well as a variety of non-malignant conditions such as bipolar disorder, myocardial infarction, obesity, renal fibrosis, rheumatoid arthritis and scar formation. However, data regarding its participation in each condition is based on few investigations. Thus, additional studies are required for verification of these results. Moreover, conduction of further association studies in different populations is necessary to validate the observed associations between miR-1908 SNPs and hematological indexes, serum metabolite levels, neuroimaging measurement, brain volume measurement, asthma and bipolar disorder.

## Future perspectives

miR-1908 is a candidate for design of novel therapeutic approaches for a wide array of human disorders. Establishment of these kinds of therapies needs comprehensive assessment of its expression in diverse steps of pathogenic events, particularly in cancers. Safety issues and effective transport of miR-1908-modulating therapies into the target cells are the main issues in this regard. These issues should be solved through application of these methods in animal models.

## Data Availability

The analyzed data sets generated during the study are available from the corresponding author on reasonable request.
